# Expression of tolerogenic dendritic cells in the small intestinal tissue of patients with celiac disease

**DOI:** 10.1016/j.heliyon.2022.e12273

**Published:** 2022-12-10

**Authors:** Farzaneh Kheiri, Mohammad Rostami-Nejad, Davar Amani, Amir Sadeghi, Afshin Moradi, Elham Aghamohammadi, Amirhossein Sahebkar, Mohammad Reza Zali

**Affiliations:** a Department of Immunology, School of Medical Science, Shahid Beheshti University of Medical Sciences, Tehran, Iran; b Gastroenterology and Liver Diseases Research Center, Research Institute for Gastroenterology and Liver Diseases, Shahid Beheshti University of Medical Sciences, Tehran, Iran; c Taleghani Hospital, Pathology Department, Shahid Beheshti University of Medical Sciences, Tehran, Iran; d Basic and Molecular Epidemiology of Gastrointestinal Disorders Research Center, Research Institute for Gastroenterology and Liver Diseases, Shahid Beheshti University of Medical Sciences, Tehran, Iran; e Biotechnology Research Center, Pharmaceutical Technology Institute, Mashhad University of Medical Sciences, Mashhad, Iran; f Applied Biomedical Research Center, Mashhad University of Medical Sciences, Mashhad, Iran; g Department of Biotechnology, School of Pharmacy, Mashhad University of Medical Sciences, Mashhad, Iran

**Keywords:** Celiac disease, Gene expression, Dendritic cells, Indoleamine 2,3-dioxygenase, Cluster of differentiation markers

## Abstract

Tolerogenic dendritic cells (tolCDs) play an important role in the regulation of inflammation in autoimmune diseases such as celiac disease (CeD). Dendritic cells express CD207, CD11c, and CD103 on their surface. In addition to the receptors mentioned above, tolCDs can express the immune-regulating enzyme indoleamine 2,3-dioxygenase (IDO). This study aimed to determine the mRNA and protein expression of CD11c, CD103 and CD207 markers, and also IDO gene expression in intestinal tissues of CeD patients in comparison to the healthy individuals. Duodenal biopsies were collected from 60 CeD patients and 60 controls. Total RNA was extracted and gene expression analysis was performed using Real-time PCR SYBR® Green method. Additionally, biopsy specimens were paraffinized and protein expression was evaluated using immunohistochemistry (IHC) for expression of CD11c+, CD207+and CD103+. Gene expression levels of CD11c (P = 0.045), CD103 (P < 0.001), CD207 (P < 0.001) and IDO (P = 0.01) were significantly increased in CeD patients compared to the control group. However, only CD103 protein expression was found to be significantly higher in CeD patients in comparison to the control group (P < 0.001). The result of this study showed that the expresion levels of CD11c, CD103, CD207 and IDO markers were higher in CeD patients compared to the controls, indicating the effort of dendritic cells to counterbalance the gliadin-triggered abnormal immune responses in CeD patients.

## 1 Introduction

Celiac disease (CeD) is an autoimmune enteropathy that is triggered by gluten ingestion in genetically susceptible individuals. It affects around 1% of the general population worldwide (Taraghikhah et al., 2020; Ludvigsson et al., 2015). Its diagnosis is aided by serological tests (most importantly the anti-tissue transglutaminase IgA antibody) and duodenal biopsy evaluation (Ashtari et al., 2019; Ehsani-Ardakani et al., 2013; Rostami Nejad et al., 2012).

Dendritic cells (DCs) are significant players in the pathogenesis of CeD. DCs have at least two main functions in CeD pathogenesis. First, they act as antigen presenting cells (APCs) involved in the presentation of gliadin peptides to mucosal CD4 + T cells. Second, they may take part in the persistence of the inflammatory responses by interacting with lamina propria (LP) T cells. Moreover, these cells have a critical role in immune response regulation in CeD by controlling other immune cells such as T and B cells (Coombes and Powrie 2008; Kim and Diamond 2015; Rescigno and Di Sabatino 2009). Some types of DC subsets which regulate tolerance are present in the lamina propria of the small and large intestines, the isolated lymphoid follicles (ILF), the Peyer patches (PPs) and the mesenteric lymph nodes (MLNs) (Matteoli et al., 2010; Rescigno and Di Sabatino 2009). Among these, tolerogenic DCs (tolDCs) are involved in regulating immune responses, maintaining homeostasis, and inhibiting inessential inflammation (Worbs, Hammerschmidt, and Förster 2017). The density of CD11c+CD103+ tolDCs is increased in CeD and these cells can release indoleamine 2,3-dioxygenase (IDO) as a mechanism to induce a tolerogenic state (Matteoli et al., 2010; Welty et al., 2013). IDO is a regulatory enzyme and has high expression in intestinal tolDCs (Coombes et al. 2007).

Langerin was thought to be expressed only in the Langerhans cells (LC), but now it is known that DCs also express the langerin. DCs can sense antigens and regulate immune responses *via* langerin (CD207). CD207+ DCs in gut-associated lymphoid tissue (GALT) maintain a balance between inflammation and immune system suppression (Chang and Kweon 2010; Figdor et al. 2002; Valladeau et al., 2000). There is scant information about the expression of tolDC-related genes and proteins in Iranian CeD patients. Therefore, this study aimed to investigate the expression of CD11c, CD103, CD207, and IDO genes, as well as the CD11c, CD103 and CD207 protein levels in the small intestine of CeD patients compared to healthy individuals.

## 2 Material and methods

### 2.1 Study population

In this study, 60 treated CeD patients (under gluten free diet (GFD) for more than 2 years) and 60 matched control subjects were recruited during 2020–2021. Celiac disease was confirmed according to the standard ESPGHAN criteria using serological tests (serum IgA tTG antibody [tTGA] and/or anti-endomysial antibody [EMA IgA]) and pathology examination according to the Marsh classification (Fuchs et al., 2019; Husby et al., 2020). Controls were subjects who had no current or prior history of CeD up to their first-degree relatives with negative serological antibodies for CeD, that attended the endoscopy unit because of digestive problems like dyspepsia or reflux but had normal upper endoscopy.

A diagnosis of patients with Marsh I/II was confirmed when the subsequent conditions were fulfilled: (1) 10 times higher serum CD antibodies (2) the presence of HLA-DQ2/8 genotyping, and (3) sustained improvement of symptoms and serological response to the GFD.

The study was approved by the Ethical Committee at the Research Institute for Gastroenterology and Liver Diseases, Shahid Beheshti University of Medical Sciences (IR.SBMU.MSP.REC.1397.615). All the patients were requested to carefully read and sign an informed consent. No financial burden related to the study was imposed on the participants.

### 2.2 Sample collection

In this study, intestinal biopsy samples were collected from the distal duodenum of cases and controls by gastroduodenoscopy. Two specimens were used for morphological and immunohistochemistry examinations and the third specimen was used to extract total RNA. Table 1 summarized the demographic data of participants in this study.

**Table 1:** Demographic data of the persons studied.

Study group	Number of persons	Age^1^ (yr)	Male		Female	
			N (%)	Age (yr)	N (%)	Age (yr)
**CeD**	**60**	**40.35 ± 12.25**	**23 (38.3)**	**41.26 ± 11.65**	**37 (61.7)**	**39.78 ± 12.72**
**Control**	**60**	**35.6 ± 13.02**	**27 (45)**	**38.7 ± 14.74**	**33(55)**	**33.06 ± 11**
**Total**	**120**	**37.98 ± 12.81**	**50 (41.66)**	**39.88 ± 13.34**	**70 (58.33)**	**37.57 ± 12.47**

1 Value is the mean ± SD. CeD: Celiac disease.

### 2.3 RNA extraction, cDNA synthesis, and RT-qPCR

A commercial Kit (RNA Extraction kit, Yekta Tajhiz Azma, Tehran, Iran) was used to extract total RNA from fresh small intestinal biopsies. RNA extraction efficiency was evaluated by Nanodrop spectrophotometer (optical density = 260/280). Total RNA was reverse-transcribed into cDNA using the RevertAid First Strand cDNA Synthesis Kit (Thermo Fisher Scientific, Inc. MA, USA). The synthesized cDNA was assessed by quantitative polymerase chain reaction (qPCR) with SYBR Master Mix (Takara Bio SYBR Premix Ex Taq (Tli RNase H Plus); Takara Bio Inc., Japan). Primer sequences were designed by Gene runner software and are presented in Table 2. Beta2 microglobulin (β2m) was used as the reference housekeeping gene. The real-time qPCR was performed using the Rotor-Gene Q MDX with the following conditions: denaturation step (10 s at 95C), annealing (30 s at 60C), followed by extension (40 s at 72C). The relative gene expression was applied by (2^−ΔΔCq^) method as follows (Livak and Schmittgen 2001):

Relative gene expression 2^−ΔΔCq^. ΔΔCq = Δcq_case_ − ΔCq_control_.Δcq_target_ − Δcq _β2m_

**Table 2:** Forward and reverse sequences of IDO, CD11c, CD103, CD207, and Β_2_m primers.

Primer	Forward primer sequence (5ʹ- 3ʹ)	Reverse primer sequence (5ʹ- 3ʹ)	Product length (bp)
IDO	GCCTCCTATTTTGGTTTATGC	CACCAATAGAGAGACCAGGA	153
CD11c	AAGAACTGTGGAGCCGACC	CCCGTCATTCCACACCATCA	129
CD103	TAGGACTGCGAGGGAACTG	TCTCCAACCGTGCNCTTCCA	142
CD207	CAACAATGCTGGGAACAATGA	CTGTCCTGTCACGGTTCTG	138
β2m	CCAGCGTACTCCAAAGATTC	ATGTCGGATGGATGAAACCC	102

IDO: Indoleamine 2, 3-dioxygenase; β2m: Beta2 microglobulin.

### 2.4 Immunohistochemistry for CD11c, CD103 and CD207

The paraffin-embedded duodenal biopsy specimens were deparaffinized in xylene, followed by rehydration by transfer through graded alcohols. For immunohistochemical analysis the following ready-to-use antibodies (Master Diagnostica, Spain) including monoclonal rabbit anti-human CD11c (Clone EP157monoclonal rabbit anti-human CD103 (Clone EP206) and monoclonal rabbit anti-human Langerin CD207 (Clone EP15863) were used. Immunohistochemical staining was performed using the Master polymer plus detection system (peroxidase) (Master Diagnostica, Spain).

Sections were HIER (heat-induced epitope retrieval) – used EDTA buffer (Master Diagnostica, Spain) pH = 8 for CD11c and CD103 and PH = 9 for CD207, 20 min at 95 °C. Then rinsed with 3–5 changes of deionized water followed by cooling at room temperature (RT) for 20 min.

Subsequently, the sections were pretreated with two drops (100 μL) of Peroxidase Blocking Reagent for 10 min at room temperature and in darkness (Master Diagnostica, Spain). Rinsed the slides in TBS 3 times for 5 min (Master Diagnostica, Spain).

The sections were incubated with monoclonal rabbit anti-CD11c antibody for 30 min at RT. For detection, we used Master Polymer plus Detection System (HRP) (Master Diagnostica, Spain) according to the manufacturer's instructions. Sections were counterstained with hematoxylin (Merck, Darmstadt, Germany) for 1–2 min finally mounted slides covered by Entellan (Merck, Darmstadt, Germany).

We also used tissue sections human tonsil sections, T lymphocytes of the small intestine mucosa and human skin as positive controls for CD11c CD103 and CD207 markers respectively.

For quantification, first, the cells positive for CD11c CD103 CD207 markers were counted in 5 different microscopic fields in both Lamina Propria and Epithelium sections, and then the mean numbers were reported (Vorobjova et al., 2015).

### 2.5 Statistical analysis

For Statistical analysis, the Mann-Whitney *U* test or independent *t*-test, and analysis of variance or Kruskal-Wallis (followed by *post-hoc* pairwise comparisons) tests were performed using the Statistical Package for the Social Sciences (SPSS) version 21.0 (IBM Corp., Armonk, NY, USA) software. The results of the different study groups are presented as mean ± SE. Parametric Pearson and non-parametric Spearman's correlation test were used for correlation between the variables. GraphPad Prism version 6.0 (GraphPad Software, Inc., La Jolla, CA, USA) was used for development of the graphs. P value less than 0.05 were considered statistically significant. Sensitivity, specificity, and receiver operating characteristic (ROC) curves with the areas under the curve (AUC) were calculated for different DC markers.

## 3 Results

### 3.1 Demographic and clinical characteristics

The CeD group consisted of 60 treated patients (mean age of 40.35 ± 12.25 years) including 23 males (38.3%) and 37 females (61.7%). Sixty age-matched non-CeD subjects (mean age of 35.6 ± 13.02 years) including 33 males (55%) and 27 females (45%) were also considered as a control group (Table 1).

Among gastrointestinal symptoms, the most common symptoms were bloating (61.7%), abdominal pain (51.7%), diarrhea (46.7%), and the most common non-gastrointestinal symptoms were fatigue (70%), anemia (48.3%), weight loss (48.3%). Based on pathology reports of the small intestinal biopsy specimens, most of the patients were classified as Marsh III (49.1%).

### 3.2 Real time PCR results

**Figure 1:**
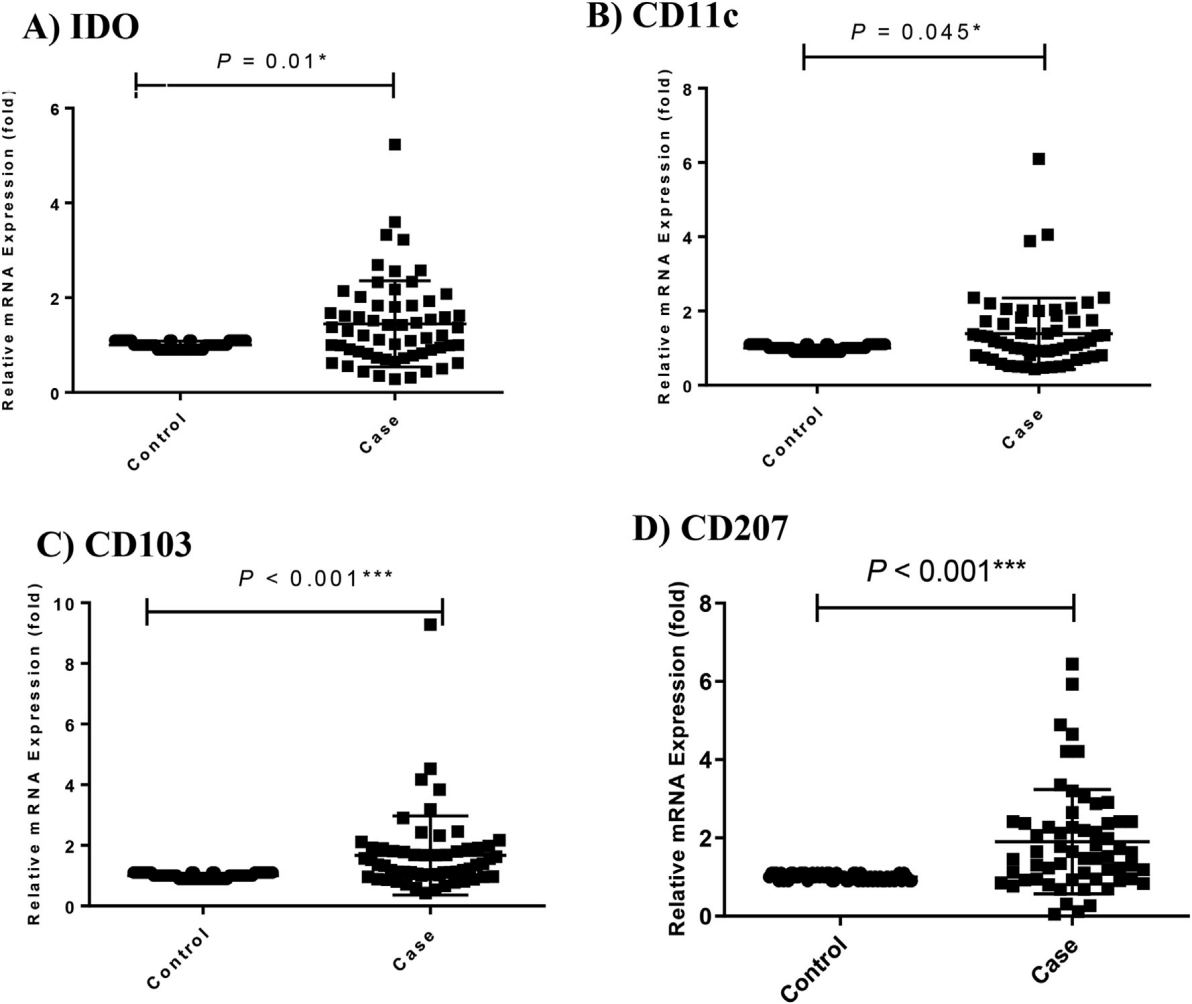
Genes expression analysis in CeD patients compared to control group. IDO mRNA expression (A); CD11c mRNA expression (B); CD103 mRNA expression (C); CD207 mRNA expression (D). Statistical analysis: Kruskall–Wallis anova and Man Whitney u. ∗, ∗∗, ∗∗∗P value is significant IDO: Indoleamine 2,3-dioxygenase.

Real-time qPCR analysis showed that the mRNA expression of IDO, CD11c, CD103 and CD207 in intestinal tissue specimens of CeD patients were significantly increased in comparison to the healthy individuals (Figure1A, B, C, D; P = 0.01, P = 0.045, P < 0.001 and P < 0.001, respectively). Fold changes of IDO, CD11c, CD103, and CD207 were 0.448, 0.388, 0.669, and 0.905 respectively (Figure1A, B, C, D).

### 3.3 Immunohistochemistry results

**Figure 2:**
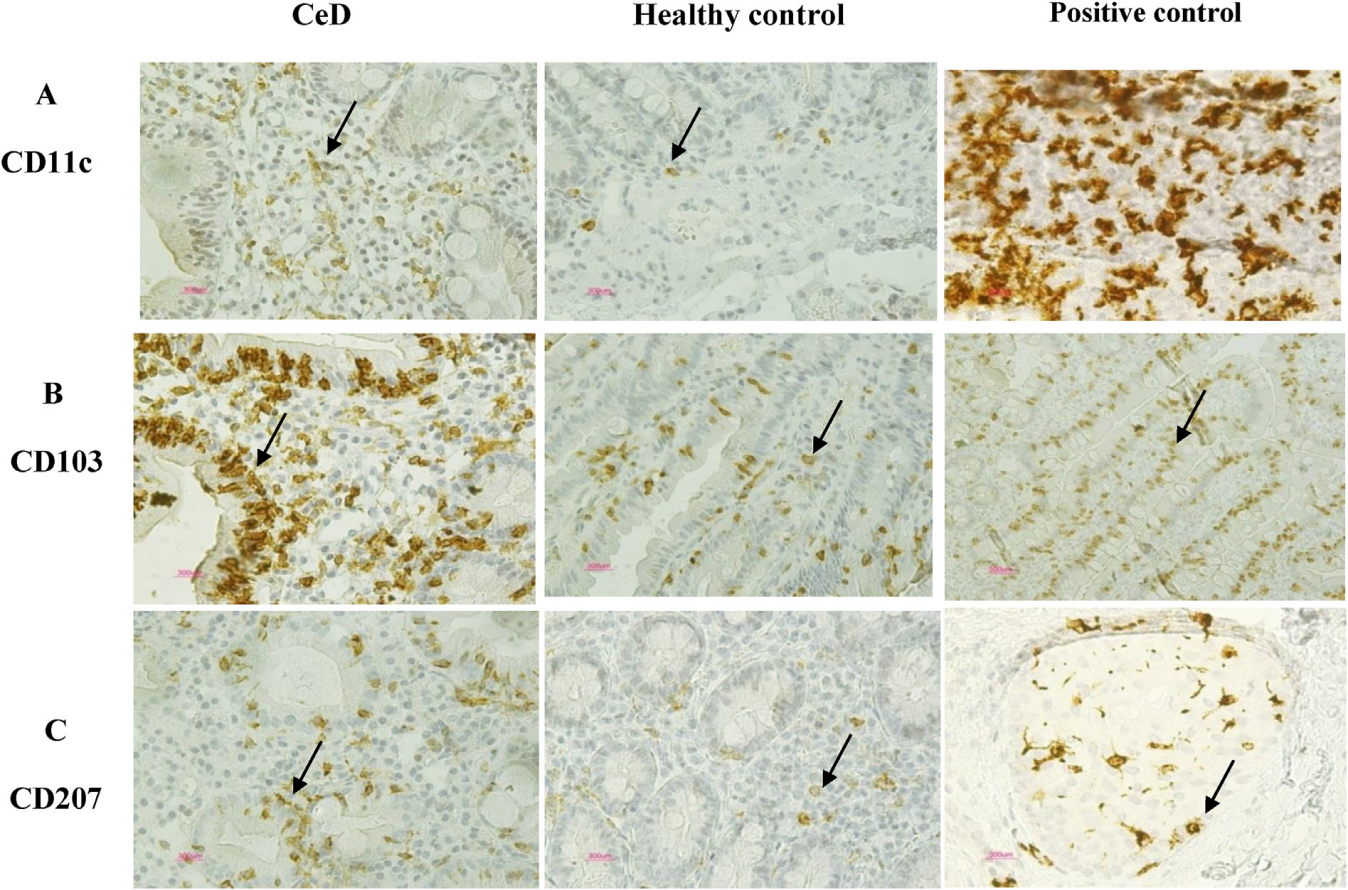
Analysis of (A) CD11c, (B) CD103, and (C) CD207 protein expression in the small intestinal tissue by immunohistochemistry. Positive staining for CD11c in the human tonsil tissue, CD103 in the T lymphocytes of the small intestine mucosa, CD207 in the human skin tissue. Indirect method. Original magnification, 40× objective; 10× eyepiece.

**Figure 3:**
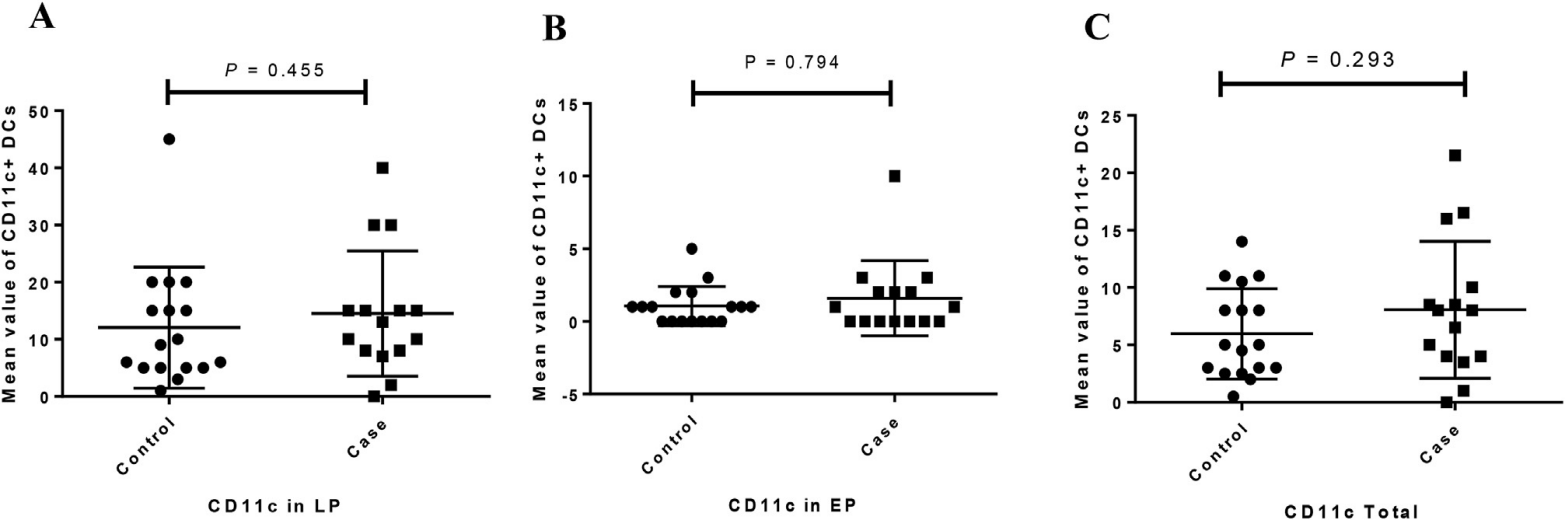
The mean values of CD11c expression in the studied groups ((A) LP expression, (B) EP expression, (C) total expression). Columns represent the mean values of positively stained cells per microscopic field in patients with CeD *vs* healthy controls. Statistical analysis: Kruskall–Wallis anova and Man Whitney u. LP: lamina propria; EP: Epithelial layer.

**Figure 4:**
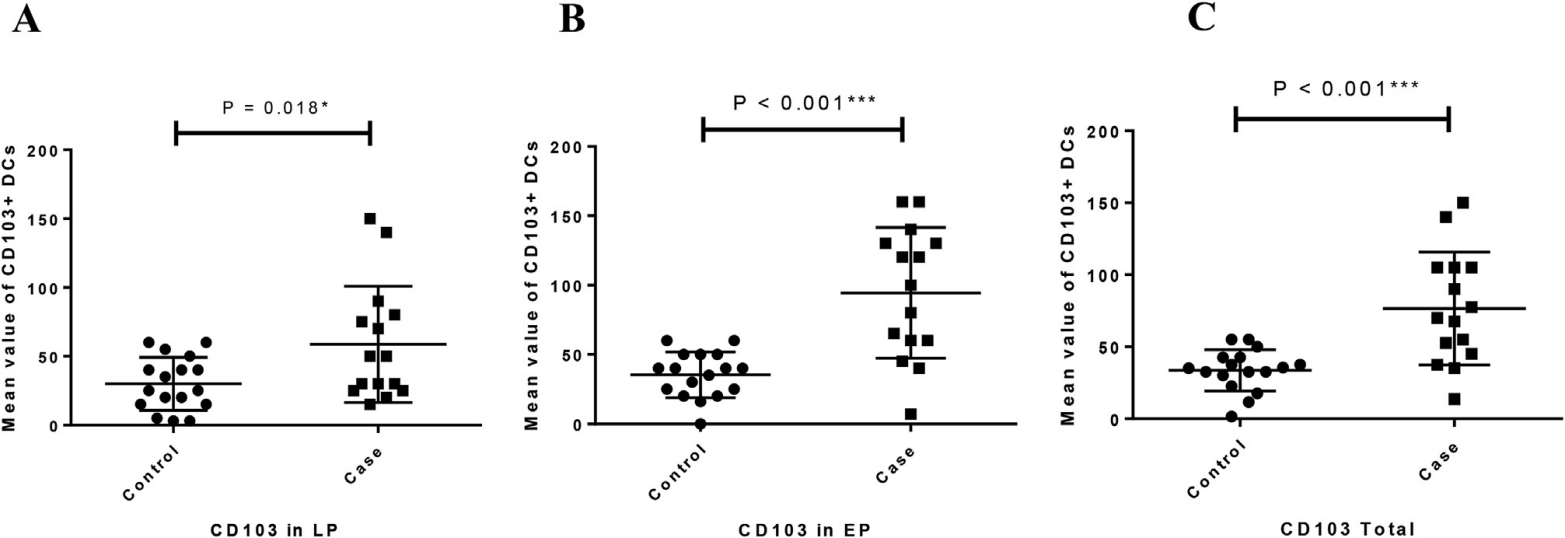
The mean values of CD103 expression in the studied groups ((A) LP expression, (B) EP expression, (C) total expression). Columns represent the mean values of positively stained cells per microscopic field in patients with CD *vs* healthy controls. Statistical analysis: Kruskall–Wallis anova and a one-way ANOVA tests. LP: lamina propria; EP: Epithelial layer. ∗, ∗∗, ∗∗∗*P* value is significant.

**Figure 5:**
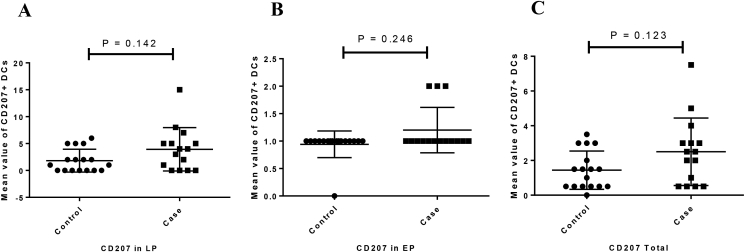
The mean values of CD207 in the studied groups ((A) LP expression, (B) EP expression, (C) total expression). Columns represent the mean values of positively stained cells per microscopic field in patients with CD *vs* healthy controls. Statistical analysis: Kruskall–Wallis anova and Man Whitney u tests. LP: lamina propria; EP: Epithelial layer.

The expression of CD11c protein in both LP (P = 0.455) and EP (P = 0.794) regions was increased in CeD patinets relative to the controls but this difference was not statistically significant (Figure 2A, Figure 3). Moreover, CD103 protein expression was significantly increased in LP and EP regions of CeD patients than in controls (P = 0.018 and P < 0.001, respectively) (Figure 2B, Figure 4). The CD207 protein expression was increased in both LP and EP regions of CeD patients compared to the controls but this difference was statistically insignificant (P = 0.142 and P = 0.246 respectively) (Figure 2C, Figure 5). A positive staining control was always considered (Figure 2).

### 3.4 Correlation analysis

According to the Mann-Whitney U test, the mRNA expression level of IDO (P = 0.042) and the protein expression levels of CD11c (P = 0.021) and CD103 (P = 0.001) were significantly higher in females patients with celiac disease compared with males.

**Figure 6:**
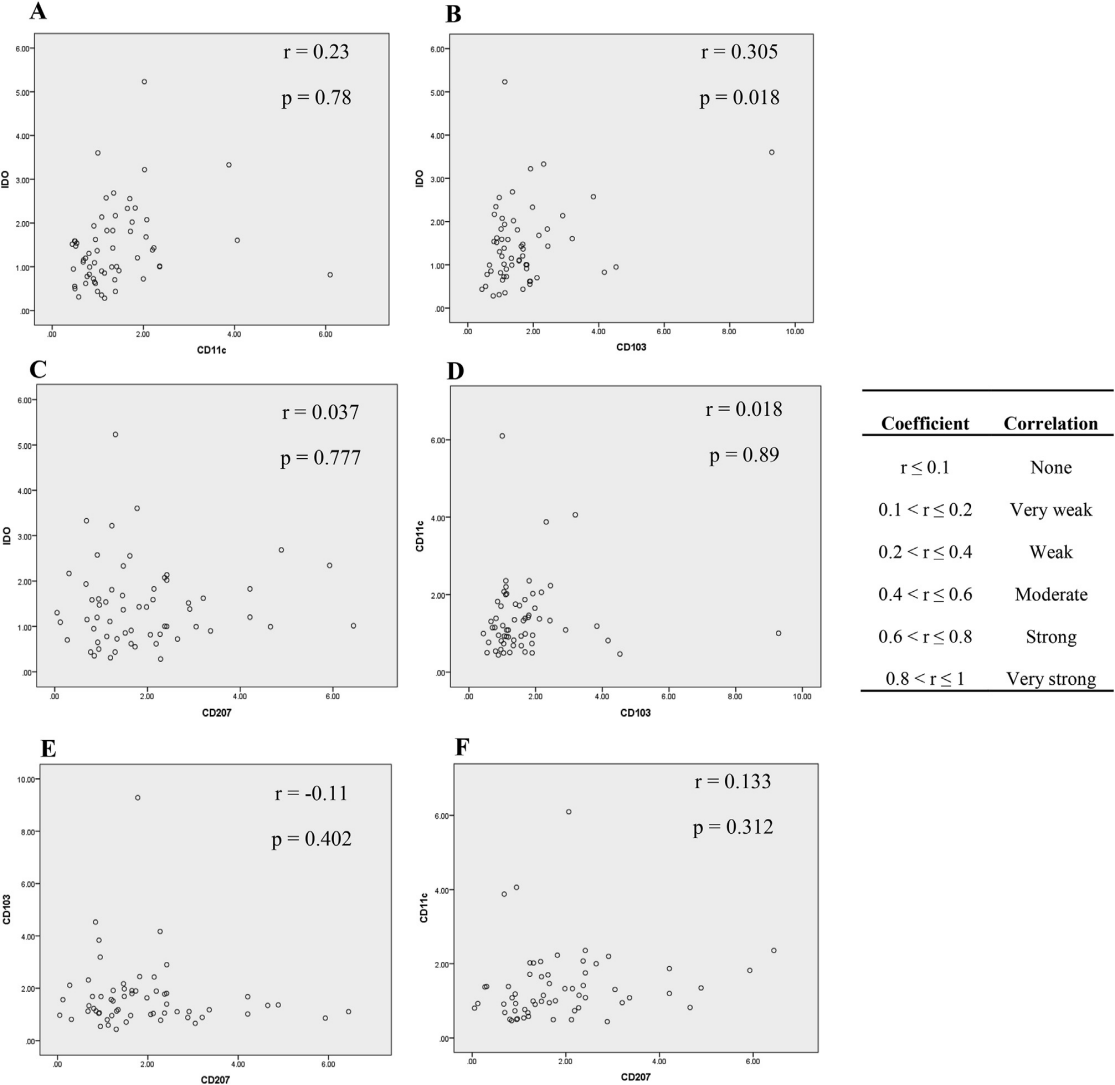
Correlations between mRNA expressions of (A) IDO and CD11c, (B) IDO and CD103, (C) IDO and CD207, (D) CD11c and CD103, (E) CD103 and CD207, (F) CD11c and CD207. The letter “r” represents correlation coefficient between two variables. P value <0.05 was considered as statistically significant. IDO: Indoleamine 2, 3-dioxygenase.

**Figure 7:**
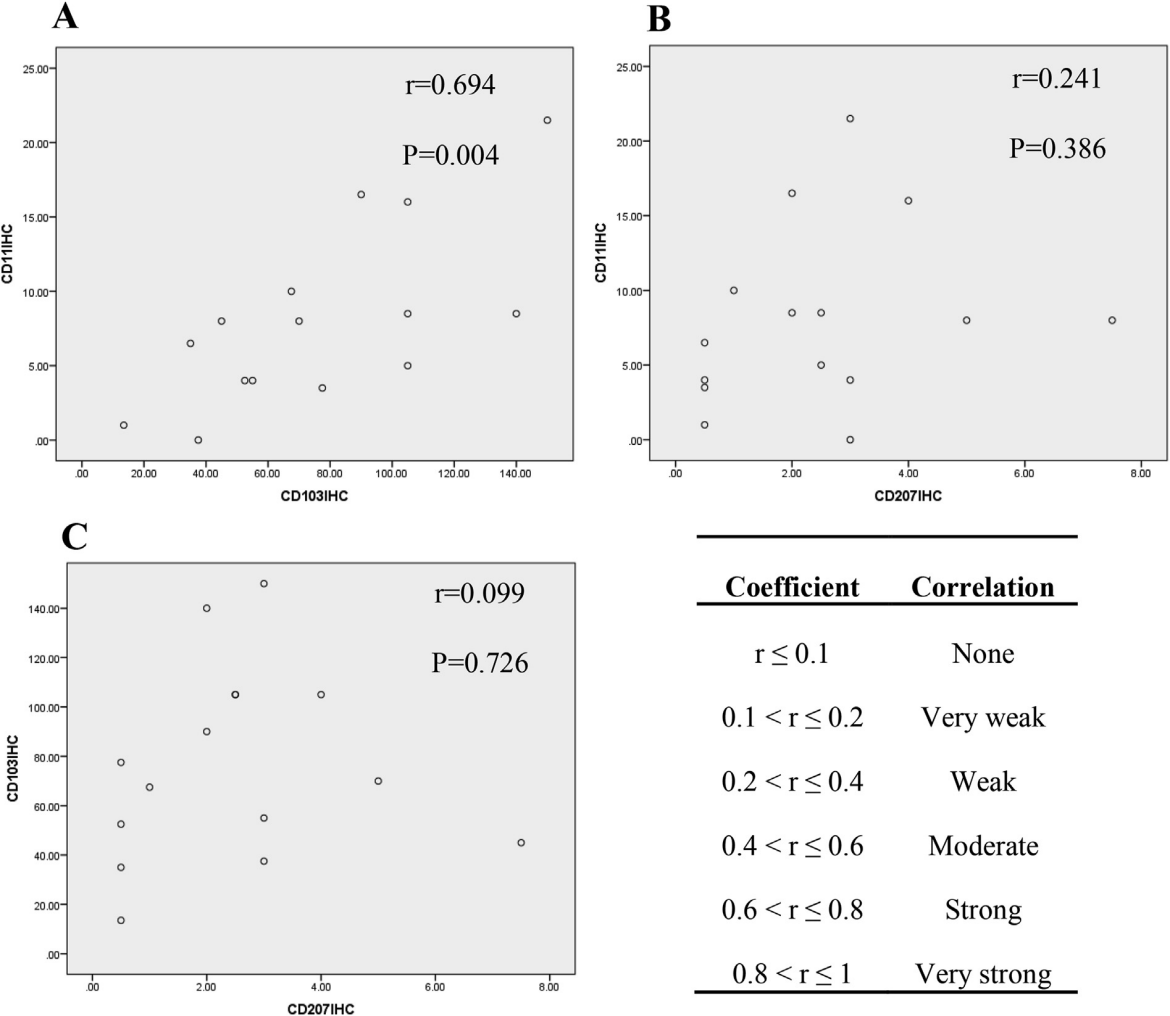
Correlations between proteins expressions of (A) CD11c and CD103, (B) CD11c and CD207, and (C) CD103 and CD207 were examined by Pearson's correlation test. The letter “r” represents correlation coefficient between two variables. P value <0.05 was considered as statistically significant.

As illustrated in Figure 6, the mRNA expression of IDO showed a significant weak correlation with CD103 mRNA level (r = 0.305; P = 0.018) and as shown in Figure 7 the protein expression of CD11c showed a strong correlation with CD103 protein level (r = 0.694, P = 0.004). According to Bivariate Spearman's correlation test analysis, osteoporosis (r = 0.337; P = 0.008) and abortion (specific to female subjects) (r = 0.329; P = 0.01) showed significant weak correlation with CD11c mRNA expression in CeD patients. Osteoporosis had also a significant weak correlation with CD11c protein expression (r = 0.365, P = 0.04) in celiac subjects. Moreover, CD103 protein expression showed significant strong correlation with diarrhea (r = −0.614, P = 0.001), moderated correlation with nausea and vomiting (r = -0.443, P = 0.011), and weak correlation with anemia (r = −0.371, P = 0.036) in CeD patients. Diarrhea (r = −0.399, P = 0.024) and abortion (r = 0.355, P = 0.046) had significant weak correlation with CD207 protein expression in CeD patients too.

### 3.5 Sensitivity and specificity of IDO, CD103, CD11c, CD207 genes and proteins in duodenal tissue as biomarkers

**Figure 8:**
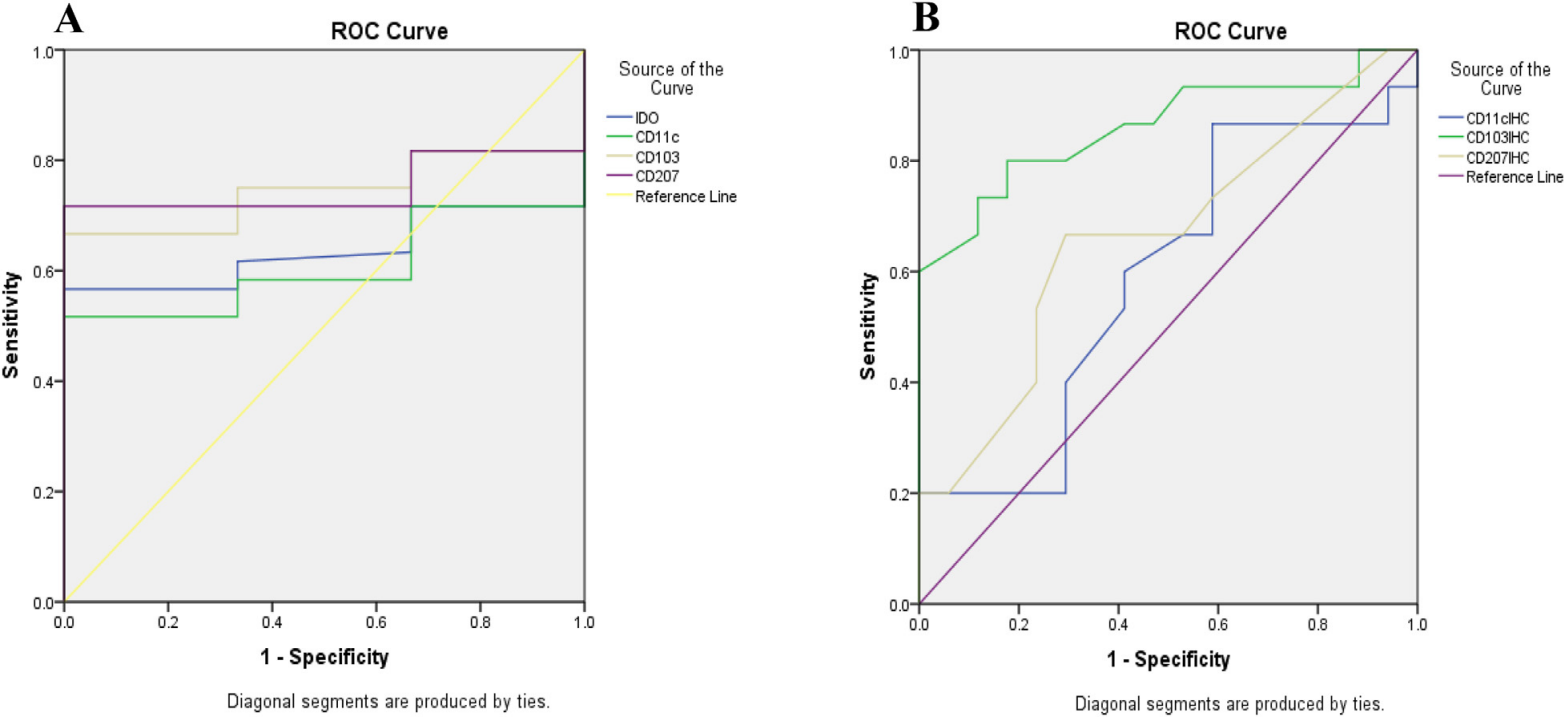
Receiver operating characteristic curves of dendritic cell genes (A) and proteins (B), showing their overall capacity to discriminate between individuals with and without the disease. The diagonal line represents a worthless test (sensitivity = 1 - specificity). The further the curve is from the diagonal line and the closer it is to the upper left-hand corner of the graph, the better the discriminative power of the test. CD: Celiac disease; IDO: Indoleamine 2, 3-dioxygenase; ROC: Receiver operating characteristic.

IDO, CD103, CD11c, CD207 genes and proteins of DCs were analyzed as potential biomarkers, using the ROC curve analysis. Results showed that, IDO (AUC = 0.64) and CD11c (AUC = 0.61) genes were poor; while CD103 (AUC = 0.74) and CD207 (AUC = 0.75) genes were relatively good biomarkers (Figure 8A and Table 3). Moreover CD11c (AUC = 0.59) and CD207 (AUC = 0.66) proteins were poor biomarkers but CD103 protein was a good biomarker (AUC = 0.86) (Figure 8B and Table 3).

**Table 3:** Areas under the curve values and the cut-off, sensitivity, and specificity values for genes and proteins dendritic cells by qRT-PCR and Immunohistochemistry on paraffin-embedded specimens.

Markers	AUC	Cut-off	Sensitivity	Specificity	P value
IDO (gene)	0.64	1.10	0.567	1	0.01
CD11c (gene)	0.61	1.12	0.51	1	0.046
CD103 (gene)	0.74	1.10	0.667	1	P < 0.001
CD207 (gene)	0.75	1.10	0.717	1	P < 0.001
CD11c (protein)	0.59	3.2500	0.867	0.412	0. 4
CD103 (protein)	0.86	43.75	0.800	0.824	P = 0.001
CD207 (protein)	0.66	1.750	0.667	0.706	P = 0.12

AUC: Area under the receiver operating characteristic curve; Cut-off: Marker value at which sensitivity and specificity are optimal.

AUC can be interpreted as follow: 90–100 = excellent; 80–90 = good; 70–80 = fair; 60–70 = poor; 50–60 = fail (18).

## 4 Discussion

In the present study, we evaluated the mRNA expression levels of IDO, CD11c, CD103 and CD207 as well as protein levels of CD11c, CD103, and CD207 in CeD patients and control subjects. Our findings showed that the mRNA expression levels of IDO, CD11c, CD103, CD207 and protein level of CD103 were significantly increased in CeD patients compared to the healthy individuals. TolDCs have various CD markers and we selected IDO, CD11c, CD103, and CD207, as markers involved in the regulation of immunological functions (Badami et al., 2011; Beitnes et al., 2011, 2012; Larsen et al., 2015; Ráki et al., 2006; Torres et al., 2007; Vorobjova et al., 2015, 2016, 2017).

In the study by Vorobjova et al., 78 small intestinal biopsy specimens from 23 children with CeD patients, 15 patients with type 1 diabetes and CeD, and 39 controls were collected and gene and protein expression profiles were evaluated using RT-PCR and IHC techniques. Similar to our results, the researchers found that the expression levels of CD103 and IDO genes and IDO protein levels were increased in CeD patients compared to controls (Vorobjova et al., 2017). In the current study, CD103 and IDO gene expression were significantly increased. In our study, the sample size was larger and most subjects were adults.

Larsen et al. investigated the effect of gluten on DCs in female BALB/c and NOD mice. They found that the expression of the CD11c gene was significantly increased in the lymphatic tissue of gluten-free diet (GFD) NOD and BALB/c mice compared to mice receiving standard gluten-containing diet (STD). Moreover, they concluded that the percentage of CD11c+ CD103+ DCs in GFD mice was increased in other tissues including liver, spleen, Peyer's patches pancreas draining lymph nodes, auricular lymph nodes and mesenteric lymph node of BALB/c and NOD mice (Larsen et al., 2015). Farache et al. found that DCs are present in different regions of the intestine (LP and EP) (Farache et al., 2013). Our finding confirmed the presence of CD11c+ CD103+ CD207 DCs in LP and EP of CeD patients. Farache et al. investigation was conducted on mice specimens and in accordance with our results, increased expression of CD11c and CD103 following adherence to a gluten-free diet was reported. Hence, it is suggested that following a gluten-free regimen is likely to result in the increased number of CD11c^+^ CD103^+^ DCs.

Different *in vitro* studies have shown that affecting human DCs by gliadin peptides increases their expression of mature and activated markers (Nikulina et al., 2020; Palová-jel'inková et al., 2019). Other *in vivo* studies are in line with these studies as well (Larsen et al., 2015; Ráki et al., 2006).

By evaluating DC diagnostic markers in 26 intestinal biopsy specimens of treated and untreated CeD patients in comparison with healthy individuals, Ráki et al. found that CD11c + DQ2 + DCs had a significant increase in treated CeD patients and healthy individuals compared to untreated CeD patients (Ráki et al., 2006). This finding is in agreement to our results.

Badami et al., (2011) assessed intestinal biopsy and peripheral blood samples of 10 T1D, 20 CeD and 16 healthy subjects for evaluating the regulatory functions of CD11c + CD103 + LP DCs. They found that the percentage of LP CD103 + CD11c + DCs was not different in the studied groups (It is noteworthy that their patients' groups were not sole celiacs). In contrast to their results, we reported an increased expression of CD11c and CD103 genes and proteins in DCs of CeD patients in LP compared to controls.

Beitnes et al. studied 16 active CeD patients and 16 controls. In contrast to our result, they showed that the density of CD103 + CD11c + DCs in the intestinal tissue samples of CeD patients was significantly decreased compared to controls. The discrepancy may be due to the low sample size of their study population.

Vorobjova et al. reported the correlation between different degrees of mucosal damage and the density of IDO + CD103 + cells. The densities of CD11c + CD103 DCs were also higher in females (Vorobjova et al., 2015). In our study, no correlation between the expression of IDO, CD11c, CD103 and CD207 genes with the severity of mucosal damage was reported. Moreover, we found that the expression of IDO genes, CD11c and CD103 proteins were higher in females.

According to Sankaran-Walters and Kovats et al., the density of intestinal DCs in women are likely higher than in men. Moreover, differentiation and functions of their DCs are regulated by estrogen receptor ligands (Kovats 2013; Sankaran-Walters et al., 2013).

In another study by Vorobjova et al., the density of CD11c and CD207 DCs was reported to be significantly increased in active pediatric CeD patients (Vorobjova et al., 2016). We similarly found that the expression of CD11c, CD207 genes was significantly higher in CeD patients compared to controls.

In conclusion, our findings showed that DCs-related markers (CD207, CD11c, CD103) and IDO expressions are increased in the small intestinal tissue of CeD patients compared to the controls. As intestinal CD103+ DCs, that express high levels of IDO, play a crucial role in mediating immune tolerance and maintenance of gut homeostasis, these observations may indicate their effort to counterbalance the gliadin-triggered abnormal immune responses in this group of patients. As the information on DCs changes during celiac disease pathogenesis is scant, our results can serve as catalysts for researchers to pay more attention to the strategies to strengthen these cells' immunomodulatory functions for finding a novel complementary therapeutic approach for celiac disease. The major limitation of the study is that our analysis was restricted to the intestinal biopsy specimens. Considering that biopsy preparation is an invasive method, it is recommended to conduct a similar study on other biological samples, including peripheral blood specimens. Studies comparing both active and treated CeD subjects (with different regimen durations) are also needed to further the results of our study.

## Declarations

### Author contribution statement

Farzaneh Kheiri: Conceived and designed the experiments; Performed the experiments; Analyzed and interpreted the data; Wrote the paper.

Mohammad Rostami-Nejad; Mohammad Reza Zali: Conceived and designed the experiments; Analyzed and interpreted the data; Contributed reagents, materials, analysis tools or data; Wrote the paper.

Davar Amani; Amirhossein Sahebkar: Conceived and designed the experiments; Analyzed and interpreted the data; Wrote the paper.

Amir Sadeghi; Afshin Moradi; Elham Aghamohammadi: Performed the experiments; Analyzed and interpreted the data; Wrote the paper.

### Funding statement

This work was supported by Research Institute for Gastroenterology and Liver Diseases, Shahid Beheshti University of Medical Sciences, Tehran, Iran (Grant No. 9398).

### Data availability statement

Data will be made available on request.

### Declaration of interest’s statement

The authors declare no conflict of interest.

### Additional information

No additional information is available for this paper.
